# Good sleep quality is associated with better academic performance among Sudanese medical students

**DOI:** 10.1186/s13104-015-1712-9

**Published:** 2015-11-23

**Authors:** Hyder Osman Mirghani, Osama Salih Mohammed, Yahia Mohamed Almurtadha, Moneir Siddig Ahmed

**Affiliations:** Internal Medicine Department, Faculty of Medicine, University of Tabuk, P.O. Box 741, Tabuk, Saudi Arabia; Faculty of Engineering and Computer Sciences, The University of Tabuk, Tabuk, Saudi Arabia; Specialist in Internal Medicine, Ministry of Health, Khartoum, Sudan

**Keywords:** Sleep quality, Academic performance, Medical students, Sudan

## Abstract

**Background:**

There is increasing awareness about the association of sleep quality and academic achievement among university students. However, the relationship between sleep quality and academic performance has not been examined in Sudan; this study assessed the relationship between sleep quality and academic performance among Sudanese medical students.

**Methods:**

A case–control study was conducted among 165 male and female medical students at two Sudanese universities. Excellent (A) and pass (C) academic groups were invited to respond to a self-administered questionnaire, using the Pittsburgh Sleep Quality Index (PSQI). Students also completed a diary detailing their sleep habits for 2 weeks prior to filling out the questionnaire. Various parameters of sleep quality were then compared between the two groups.

**Results:**

A significant difference (p < 0.001) between the excellent and average groups was found for overall sleep quality, subjective sleep rating, bedtime later than midnight, sleep latency, and daytime dysfunction (during driving, preparing a meal, etc.). No differences were found between groups for the use of sleep medications. The mean sleeping hours was (7 ± 1.9) and (6.3 ± 1.9) for the excellent and pass groups respectively (p < 0.05). A significant difference (p < 0.001) between the excellent and average groups was found for weekday and weekend bedtime, weekend wake-up time, and weekend wake-up delay. No differences were found between groups for the weekday’s wake- up time, and bedtime delay during weekends. Besides, snoring was present in 9.2 % of the excellent group versus 28 % in pass group (p < 0.005).

## Background

Physiologic and cognitive function in humans varies considerably during the day along the circadian rhythm. The sleep and wakefulness cycle is extensively regulated through intrinsic neural mechanisms (e.g., suprachiasmatic nucleus) [1]. However, the timing and expression of sleep and wakefulness are highly influenced by environmental factors [2]. Sleep deprivation of varying occasions and durations can substantially impair physical, cognitive, and emotional functions. Medical students and house medical staff work for long hours daily, and this continuous shifting without recuperation time has raised concerns about the severe effects of poor sleep quality in student training, medical errors, and patient safety [2].

Many factors alter sleep habits including coffee and tea intake, overuse of the internet and other social media, and the use of sleep medications. Furthermore, various medical problems profoundly disrupt sleep, including obstructive sleep apnea, depression, chronic sleep deprivation, narcolepsy, cataplexy, and idiopathic hypersomnia [3, 4].

While these various factors all contribute to sleep quality, the relationship of the academic achievement to sleep habits is a key issue, especially for medical students [2]. Poor sleep quality has been associated with reduced academic achievement [5], and sleep quality has also been reported to be related to negative effects on health, emotional feelings and well-being in college students [6].

Sleep quality affect medical student physical, mental health, and working capacity [7], which in turn influence the community in the form of accidents and medical error. No data are currently available regarding the effect of sleep quality on medical student performance in Sudan. Sudan is one of the largest countries in Africa, taking about 2 % of the earth’s surface, so the previous studies may not generalize to Sudan, due to environmental conditions, social conditions, and school environment. Therefore, we conducted the current study to understand the sleep habits of Sudanese medical students and whether they like those in Western countries had poor sleep quality.

In the current study, we examined the relationship between the sleep quality and academic performance among medical students by comparing groups with different levels of academic performance. Measures to improve both living condition and sleep quality are recommended.

## Subjects and methods

### Study design

This case–control study based on academic performance was conducted at two universities (University of Omdurman and Bahri) in Khartoum, Sudan.

### Subjects

This study aimed to encompass all the students in the fifth and sixth classes who score A (excellent) and C (pass) in the previous semester (they were selected from the total number of 300 medical students registered for these classes), those with score B (good) and score D (fail) were excluded from the study. One hundred sixty-five, medical students responded to the questionnaire, response rate 81.6 % (165/202). Twenty-five medical students who did not record their grades were also excluded. School performance was stratified as A, (excellent), B (good), C (pass), and D (fail). An orientation meeting was arranged with the participants, and they were orally briefed by two of the researchers (HM, and MA) about the research objectives and how to fill the questionnaire. Group leaders from all the semesters helped to distribute the questionnaire. Participation was voluntary and unpaid. All the participants signed a written informed consent form.

Subjects were asked to maintain a diary documenting sleep and wake times for 2 weeks and then they completed the Pittsburgh Sleep Quality Index (PSQI). This instrument has been previously validated for college students in sub-Saharan Africa [8] and was shown to be consistent with objective measures [9]. The PSQI contains seven components, each with a score from 0 to 3 with 3 indicating the greatest dysfunction. The global sleep quality score ranged from 0 to 21, and candidates with PSQI of more than 5 were labeled as having poor sleep quality, and those with PSQI of less than or equal 5 as good sleep quality [10].

The PSQI (http://www.sleep.pitt.edu/content.asp?id=1484&subid=2316) measures sleep-related habits in the past month including sleep latency, sleep duration, sleep efficiency, sleep disturbance, subjective sleep quality, daytime dysfunction, and sleep medication use. Sleep efficiency is the ratio of time spent in sleep (total sleep time) to the amount of time spent in bed.

The bedtime delay is the (weekday bedtime-weekends bedtime), and wake-up time delay is the (wake-up time during weekdays-wake-up time during weekends) A sleep time difference of more than an hour between weekdays and weekends was considered irregular bedtime [11].

Data were collected at times other than examination days to avoid stress during the preparation for exams. Approval for the study was provided by the ethical committees of Omdurman and Bahri Universities.

### Statistical analysis

Data were analyzed by using statistical software (SPSS version 20), Descriptive statistics was reported as means and frequencies ANOVA was used for testing the significant difference between study groups. The results were considered statistically significant when p ≤ 0.05.

## Results

165 medical students were enrolled in the study, and 25 incomplete questionnaires were not included. The two groups consisted of 65 excellent (A) and 75 average (C) grade students.

Out of 140 medical students, female dominance was evident: (72.4 %) among the excellent group, and 73.3 % among the average group, with no statistical difference between the two groups (P = 0.73). Bad sleep quality was detected in 24 (36 %) of the excellent group, and 71 (94.6 %) of the passing group, with a significant difference seen between groups (p < 0.001). Bedtime later than midnight was detected in 55.3 % in the excellent group and 85.3 % in the average group with statistical significant difference between the two groups (p < 0.001), other student’s characteristics are shown in Table 1. Table 2 illustrates sleep characteristics from the PSQI for the two groups in which, the mean age was 22.5 ± 1.8 for the excellent group and 22.6 ± 1.9 for the average group with no statistical difference between the groups (P = 0.823). The overall sleep quality was 4.03 ± 3.3 for the excellent group and 10.6 ± 3.8 for the average group with a significant statistical difference between the two groups (p < 0.001) and F = 118.2. The mean sleeping hours at night was 7 ± 1.9, among the excellent group, and 6.3 ± 1.9 among the average group, (p < 0.05) and F = 4.018. Sleep latency in minutes was 14.8 ± 15.2 in the excellent group, and 30.8 ± 28.5 in the average group (p < 0.001) and F = 15.334. Subjective sleep rating was 0.63 ± 0.92 for the excellent students and 1.5 ± 0.95 for the average students (p < 0.001) and F = 29.3. Table 3 showed weekdays and weekends bedtime and wake-up times: the excellent group weekdays and weekend bedtime were 11.54 pm ± 1.54 h and 12.54 am ± 3.36 h respectively, while in the average group they were 1.12 am ± 1.42 h and, 2.48 am ± 2.12 h respectively, with significant statistical difference between the two groups (P < 0.001) and F = 15.628 and 14.271 respectively. No significant statistical difference was evident between the two groups as regards weekdays wake- up time (P = 0.209) and F = 1.592. Regarding weekend wake-up time, it was 8.24 am ± 2.54 h in the excellent group and 10.48 am ± 3.33 h in the average group, with a significant statistical difference between the two groups (P < 0.001) and F = 19.711.

**Table 1:** Clinical characteristics of the study groups

Parameter	Excellent (%)	Pass (%)	*P* value
Sex			
Male	27.6	26.7	0.738
Females	72.4	73.3	
Bad sleep quality	36.9	94.6	0.000
Bedtime later than midnight	55.3	85.3	0.001
Snoring or coughing	9.2	28	0.003
Subjective rating of bad sleep	15.3	46.6	0.000
Daytime dysfunction	26.1	38.6	0.000
Use of sleep medications	23	22.6	0.758

**Table 2:** Characteristics of sleep from the PSQI for the two study groups

Character mean ± SD	Excellent	Average	F	Sig
Age years	22.5 ± 1.8	22.6 ± 1.9	0.050	0.823
Sleeping hours	7.00 ± 1.9	6.30 ± 2.0	4.018	0.047
Sleep latency	14.8 ± 15.2	30.3 ± 28.5	15.334	0.000
Subjective sleep rating	0.63 ± 0.92	1.5 ± 0.95	29.3	0.000
Overall sleep quality	4.03 ± 3.3	10.6 ± 3.8	118.2	0.000

**Table 3:** Variation between wake-up and bed time in weekdays and weekends between excellent and average groups

Variable	Excellent (Mean ± SD)	Average (Mean ± SD)	F	Sig
Bed time				
Weekdays	11.54 pm ± 1.54 h	1.12 am ± 1.42 h	15.628	0.000
Weekend	12.54 am ± 3.36 h	2.48 am ± 2.12 h	14.271	0.000
Bedtime delay during weekend^a^	1.2 ± 1.8	1.6 ± 1.7	1.441	0.232
Wake-up time				
Weekdays	6.54 am ± 1.48 h	7.18 am ± 2.12 h	1.592	0.209
Weekend	8.24 am ± 2.54 h	10.48 am ± 3.33 h	19.711	0.000
Wake up time delay during weekend	2.1 ± 2.0	3.8 ± 2.2	25.031	0.000

^a^Bedtime during weekend-bedtime during weekday

The bedtime delay during weekends was 1.2 ± 1.8 h in the excellent group and 1.6 ± 1.7 in the average group, with no significant difference between the groups (P = 0.232) and F = 1.441. Regarding wake-up delay during weekend it was 2.1 ± 2.0 in the excellent group, and 3.8 ± 2.2 in the average group, with statistical difference between the two groups, (P < 0.001), and F = 25.031.

## Discussion

Poor sleep quality and daytime sleepiness have been reported to be associated with poor academic performance [12], cardiovascular events, and motor vehicle accidents. This study assessed the association between sleep quality and academic performance in Sudanese medical students.

We found a significant difference between excellent and average students regarding overall sleep quality. This result is similar to the study conducted by Seblewengel et al. [8] in Ethiopia in which significant variation was found between the good sleeper and bad sleepers as regarding all components of the Pittsburg Sleep Quality Index except for sleep latency. The present study reported a significant difference between average and excellent medical student with regards to sleep latency, in contrast to the above study.

Our data showed a highly significant difference in sleep quality (as measured with PSQI) between excellent and average medical students and is in agreement with studies conducted among college students by Aluoja and Tavares [6, 13] who found a significant negative correlation between global sleep quality score and grade point average supporting the hypothesis that bad sleep quality is associated with poor academic performance.

Poor sleep is associated with lack of concentration and inability to function during the day [8] that affect academic performance. In the current study, sleep duration ranged from 3 to 8 h with a mean of 7.00 ± 1.9 in the excellent group, and 6.30 ± 2.0 in the average student group, and a significant difference was found between the two groups of medical students (P = 0.047). Similar to our result, is the study conducted in Saudi Arabia by Bahammam [14] who concluded that decreased nocturnal sleep time is negatively associated with academic performance in medical students. Furthermore Bahammam demonstrated that, late bedtime on weekdays and weekends is associated with lower academic performance, and going with the present finding in which, the excellent student weekdays bedtime was earlier than average students, 11.54 pm ± 1.54 h versus 1.12 am ± 1.42 h in average students (P < 0.001), and F = 15.628. The excellent student’s weekend’s bedtime was also earlier than the average students, 12.54 am ± 3.36 h, and 2.48 am ± 2.12 h respectively.

In the current study, students delayed their bedtime by 1.2 h in excellent students, and 1.6 h in average students, similarly a study published by Colette et al. [15] showed that university students delay their bedtime by 1.5 h.

The outstanding result of the present study is that 61.4 % of the study group had bad sleep quality and is higher than studies conducted in Malaysi [12] in which 20 % of medical students had bad sleep quality and Estonia which showed that 6 and 1 % of medical students were poor and very poor sleepers respectively [6].

Due to demands of the academic environment, most medical students are involved in the late- night study and excessive internet use and some also consume stimulants to stay awake at night [7]. The present data showed that; the majority of the average student study group (69.2 %) goes to bed later than midnight during weekdays; similarly a study conducted in Lebanon among university students and concluded that 89.9 % of the responders went to bed after 11 pm on weekdays [15].

### Limitations of the study

The current results are appropriately interpreted with certain caveats. The present study was conducted at only two colleges, so generalizability cannot be ensured; further multi- center larger studies including students with all grades focusing on those with grade D (fail) are needed. Comparison between public and private medical schools with different living environments and class timetable can also be done. The reliance on the self-administered questionnaire to collect data is subjective as students may not have accurately reported their sleep habits. Research suggests that people may overestimate or underestimate self- reported values.

Being a cross-sectional study we cannot determine a causal relationship between sleep quality and academic performance. In the present study we excluded those with grade D (failing) which is the most important group to focus on; also we were not able to control for potential confounders like stress and class attendance. Experiments conducted on humans showed that sleep deprivation leads to impairments of memory, concentration and performance [16].

Due to the growing concerns about the association of poor sleep quality with many physical and mental diseases, it is imperative to conduct additional research to examine potential causes, and implement appropriate preventive measures and treatment when needed.

## Conclusion

A strong relationship is evident between good sleep quality and high academic performance. Measures to improve the sleep quality among Sudanese medical. Students aimed at promoting living environment, providing health education on proper. Sleep hygiene and better scheduling of lectures are highly recommended.

## Abbreviations

PSQI: the Pittsburg quality index
SPSS: statistical package for the social sciences
